# Pulmonary Vasodilator Therapy Is Associated with Decreased Mortality in Patients with Chronic Lung Disease and Severe Pulmonary Hypertension

**DOI:** 10.3390/jcdd11030089

**Published:** 2024-03-08

**Authors:** Olivia Schanz, Gerard J. Criner, Parth Rali, Shameek Gayen

**Affiliations:** Department of Thoracic Medicine and Surgery, Lewis Katz School of Medicine at Temple University Hospital, Philadelphia, PA 19140, USA; olivia.schanz@tuhs.temple.edu (O.S.); gerard.criner@tuhs.temple.edu (G.J.C.); parth.rali@tuhs.temple.edu (P.R.)

**Keywords:** pulmonary hypertension, PAH-specific therapy, chronic lung disease

## Abstract

The mortality benefit of PAH-specific therapy for patients with pulmonary hypertension (PH) associated with lung disease is not clear. Our aim was to determine whether pulmonary arterial hypertension (PAH)-specific therapy is associated with reduced mortality among all patients with PH associated with lung disease and in patients with chronic lung disease and severe PH. This was a retrospective cohort study of patients at our institution with chronic lung disease and PH. Survival analysis was performed by comparing patients who received PAH-specific therapy with patients who did not receive pulmonary vasodilators in the entire cohort and in a subgroup of patients with severe PH defined as PVR > 5 WU. We identified 783 patients with chronic lung disease and PH; 246 patients met the new criteria for severe PH. In the entire cohort, a similar survival probability was seen between the treated and untreated PH groups (logrank *p* = 0.67). In the severe PH subgroup, patients treated with PAH-specific therapy had increased survival probability (logrank *p* = 0.03). PAH-specific therapy was independently and significantly associated with decreased mortality in severe PH (HR 0.31, 95% CI 0.11–0.88, *p* = 0.03). PAH-specific therapy may confer a mortality benefit in patients with chronic lung disease and severe PH, which is now defined as PVR > 5 WU, similarly to those with pulmonary arterial hypertension.

## 1. Introduction

Advanced lung disease with pulmonary hypertension (PH) portends a worse prognosis than that of patients with lung disease without PH. PH is an independent risk factor for poor survival across multiple etiologies of lung disease, including chronic obstructive pulmonary disease (COPD), interstitial lung disease (ILD), and sarcoidosis [1,2,3,4]. Despite the poor survival, treatment has remained elusive, with mixed outcomes of pulmonary arterial hypertension (PAH)-specific therapy for survival [5].

To date, much of the literature describing the effect of PAH-specific therapy on PH associated with lung disease does not distinguish outcomes by baseline PH severity. It is possible that the response to PAH-specific therapy may differ based on the magnitude of PH, a result that is lost when all patients with PH are pooled. Furthermore, the hemodynamic definition of severe PH has been called into question, making studies that examine PH associated with lung disease unreliable.

Until recently, the European Society of Cardiology (ESC) and European Respiratory Society (ERS) guidelines for severe PH in chronic lung disease defined a mean pulmonary artery pressure (mPAP) of ≥35 mmHg or an mPAP of ≥25 mmHg with a cardiac index of ≤2.5 L/min/m^2^. However, several studies have challenged whether the mPAP cut-offs inform survival [6,7,8]. In fact, the 2022 ESC/ERS guidelines for the diagnosis and management of PH changed the criteria for severe PH to a pulmonary vascular resistance (PVR) of >5 WU [9]. 

We hypothesize that PAH-specific therapy confers a mortality benefit in patients with chronic lung disease and pulmonary hypertension, particularly those with severe PH defined as PVR > 5 WU. Our objective was to determine whether PAH-specific therapy is associated with reduced mortality among all patients with chronic lung disease and PH, as well as among those with chronic lung disease and, specifically, severe PH. 

## 2. Materials and Methods

### 2.1. Study Design and Definitions

This was a retrospective cohort study of patients with chronic lung disease and PH diagnosed via right heart catheterization (RHC) from 2011 to 2023. Chronic lung disease was diagnosed via pulmonary function testing (PFT) and/or computed tomography (CT). Lung disease diagnoses included COPD, idiopathic pulmonary fibrosis (IPF), other fibrotic ILD, non-fibrotic ILD, pulmonary sarcoidosis, and combined pulmonary fibrosis and emphysema (CPFE). Those with connective tissue disease–ILD all had significant parenchymal involvement; none had PAH as the sole pulmonary manifestation of their connective tissue disease. Those with sarcoidosis all had significant parenchymal involvement with pulmonary fibrosis. PH was defined as mPAP > 20 mmHg via RHC and was stratified according to the 2022 ESC/ERS guidelines for PH. RHC was performed on baseline oxygen requirements. Patient characteristics, comorbidities, and clinical characteristics—including RHC values, spirometry, six-minute walk distance (6MWD), diffusing capacity of the lungs for carbon monoxide (DL_CO_), oxygen requirements, and brain natriuretic peptide (BNP)—at the time of PH diagnosis were collected. The outcomes of mortality without lung transplantation, lung transplant recipients, living without lung transplantation, and time to an outcome were collected as well. These variables were compared between patients with chronic lung disease and PH who received PAH-specific therapy (treated) and those who did not receive PAH-specific therapy (untreated) in the entire cohort. The outcomes were then compared between treated and untreated patients who met the new criteria for severe PH. The decision to treat PH was made at the discretion of the individual pulmonary physicians providing care for these patients. Our study met approval for a waiver of informed consent according to the Western Institutional Review Board (Protocol #29422).

### 2.2. Statistical Analysis 

Statistical analysis was performed by comparing treated PH to untreated PH in the entire cohort and then among those with PVR > 5 WU. All continuous variables were presented as the mean ± standard deviation unless stated otherwise. Categorical variables were compared using the Pearson chi-squared test or Fisher exact test where applicable. Continuous variables were compared between groups using the Mann–Whitney *U* test. Kaplan–Meier survival analysis was performed to compare the survival probability from the time of PH diagnosis to the time of the last follow-up between the treated and untreated PH groups. Univariable Cox regression was performed to determine significant associations with mortality. Variables with significant associations with an outcome (*p* < 0.05) were subsequently utilized in multivariable Cox regression to determine independent and significant associations with mortality. The statistical analysis was performed using IBM SPSS Statistics, Version 25.

## 3. Results

**Figure 1:**
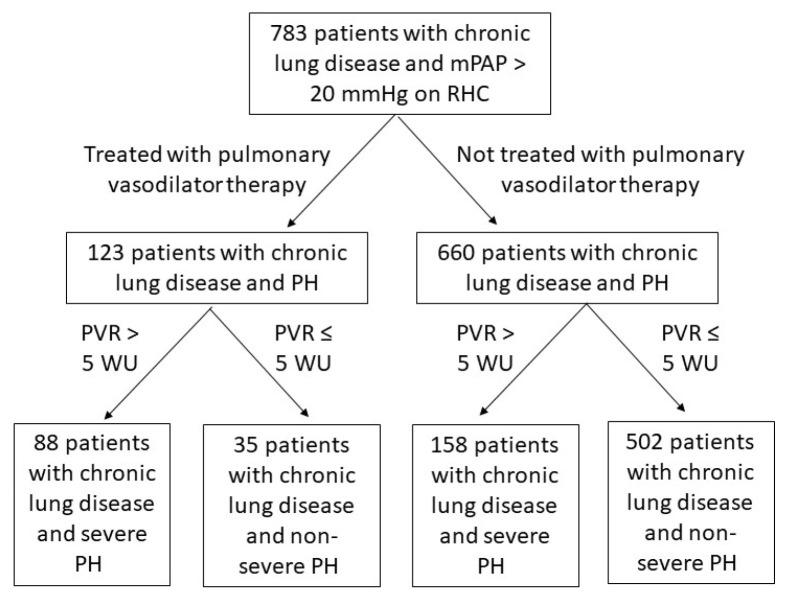
Patient cohort flowsheet. Inclusion and exclusion of patients with chronic lung disease and pulmonary hypertension: Chronic lung disease was defined as either COPD, IPF, another fibrotic ILD, non-fibrotic ILD, sarcoidosis, or CPFE. Pulmonary hypertension was defined as a mean pulmonary artery pressure of >20 mmHg upon right heart catheterization. Severe pulmonary hypertension was defined as a PVR of >5 WU. A total of 783 patients with chronic lung disease and pulmonary hypertension were included in the study. COPD: chronic obstructive pulmonary disease. CPFE: combined pulmonary fibrosis and emphysema. ILD: interstitial lung disease. IPF: idiopathic pulmonary fibrosis.

We identified 783 patients with chronic lung disease and PH (Figure 1). Of this cohort, 123 patients received PAH-specific therapy and 660 did not. The specific PAH-specific therapies prescribed are displayed in Supplementary Table S1. Among those who received PAH-specific therapy, a lower proportion of patients had COPD and a higher proportion had IPF, other fibrotic ILD, non-fibrotic ILD, and sarcoidosis compared to those who did not receive PAH-specific therapy (Table 1). Of those who received PAH-specific therapy, 23 patients had combined pre- and post-capillary PH, while the rest had pure pre-capillary PH. The majority of the combined pre- and post-capillary PH patients who received PAH-specific therapy were treated with phosphodiesterase-5 inhibitors, with one patient receiving inhaled prostacyclin and one patient receiving an endothelin receptor antagonist.

The lung function according to spirometry, DL_CO_, 6MWD, and oxygen requirements were similar between the treated PH and untreated PH groups (Table 2). Notably, the pulmonary hemodynamics were significantly more severe in the treated PH group, with a higher systolic pulmonary artery pressure (sPAP), higher mPAP, higher PVR, and lower cardiac index (CI) than those in the untreated PH group. The BNP at the time of PH diagnosis was also higher in the treated PH group (Table 2).

Those who received treatment with PAH-specific therapy had more severe pulmonary hemodynamics, as evidenced by the significantly higher sPAP, mPAP, and PVR, along with the significantly lower CI. Lung function, 6MWD, and oxygen requirements were similar between the two groups. 

**Figure 2:**
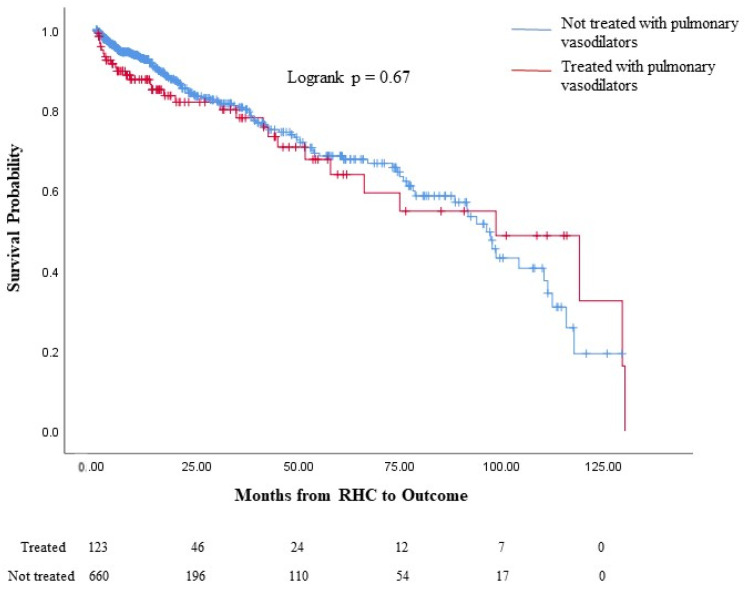
Survival analysis of those treated with PAH-specific therapy versus those who were untreated in the entire cohort. Comparison of the survival probability between the treated PH and untreated PH groups among the entire cohort with chronic lung disease and the PH cohort from the time of PH diagnosis: The number of patients in each group who were at risk is displayed on the bottom of the figure. The changes in the graph are due to death without a lung transplant while also censoring those who received a lung transplant, as they would no longer be at risk of death without a transplant; lung transplantation is not reflected in the actual Kaplan–Meier curves. In the entire cohort, no significant differences in survival probability (logrank *p* = 0.67) were seen between the treated PH and untreated PH groups. PH: Pulmonary hypertension.

Similar proportions of patients in the treated PH and untreated PH groups from the entire cohort died without a lung transplant. A larger proportion of the untreated PH group received lung transplantation, while a larger proportion of the treated PH group was alive without a lung transplant at the last follow-up; the median survival time from PH diagnosis was similar between the two groups (Table 3). The Kaplan–Meier survival analysis of the entire cohort demonstrated similar survival probabilities between the treated PH group and the untreated PH group (logrank *p* = 0.67, Figure 2).

The mortality rate was higher, though statistically similar, among those who received PAH-specific therapy than among those who did not. A significantly larger proportion of those who did not receive PAH-specific therapy received lung transplantation, while a significantly larger proportion of those who received PAH-specific therapy were alive without a lung transplant at the last follow-up.

A total of 246 patients met the newest criteria for severe PH in chronic lung disease, which was again defined as PVR > 5 WU. In this subgroup, 88 patients were treated with PAH-specific therapy, and 258 patients were not treated with PAH-specific therapy (Table 4). The clinical characteristics, including the lung function, DL_CO_, 6MWD, RHC values, and hypoxemia, were similar between the treated PH and untreated PH groups among those with severe PH. A significantly larger proportion of the untreated severe PH group had an elevated BNP compared to the treated severe PH group (Table 4).

**Figure 3:**
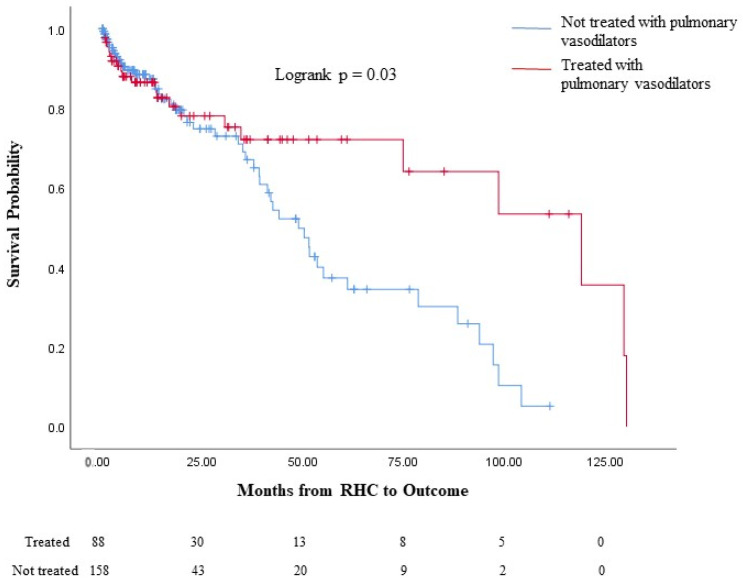
Survival analysis of patients treated with PAH-specific therapy versus untreated patients in the chronic lung disease and severe pulmonary hypertension subgroup. Comparison of survival probability between patients with treated PH and untreated PH among those with chronic lung disease and severe PH (PVR > 5 W’U) from the time of PH diagnosis. The numbers of patients who were at risk in each group are displayed at the bottom. Changes in the graph are due to death without a lung transplant while also censoring those who received a lung transplant, as they would no longer be at risk of death without a transplant; lung transplantation is not reflected in the actual Kaplan–Meier curves. In the cohort with chronic lung disease and severe PH, reduced survival probability (logrank *p* = 0.03) was seen among those who were not treated with PAH-specific therapy. PH: Pulmonary hypertension. PVR: Pulmonary vascular resistance.

Though not statistically significant, a larger proportion of untreated severe PH patients than treated severe PH patients died without lung transplantation (Table 5). Similarly to the entire cohort, a significantly larger proportion of untreated severe PH patients underwent lung transplantation, while a significantly larger proportion of the treated severe PH group was alive without lung transplantation at the last follow-up. Notably, the median survival time among those with severe PH was significantly longer in the treated PH group (Table 5). The Kaplan–Meier survival analysis for the chronic lung disease and severe PH subgroup demonstrated increased survival probability from the time of RHC diagnosis of PH among those receiving PAH-specific therapy compared to that of those who did not receive PAH-specific therapy (logrank *p* = 0.03, Figure 3).

When analyzing those with severe PH (PVR > 5 WU), the mortality rate was actually lower among those who received PAH-specific therapy than among those who did not, though not significantly so. A significantly larger proportion of those who did not receive PAH-specific therapy received lung transplantation, while a significantly larger proportion of those who received PAH-specific therapy were alive without a lung transplant at the last follow-up. The median time for survival was significantly prolonged among patients with severe PH receiving PAH-specific therapy compared to that among patients with severe PH who did not receive PAH-specific therapy. 

Multivariable Cox regression was performed among those with chronic lung disease and severe PH to test for significant and independent associations with mortality (Table 6). Treatment with PAH-specific therapy, underlying lung disease, functional vital capacity (FVC), severely reduced DL_CO_, hypoxemia, and elevated BNP (defined as >100 pg/mL) were included in the regression model; pulmonary hemodynamic parameters were not, as all patients in this analysis had severe PH and similar pulmonary hemodynamics across the treated and untreated groups. When accounting for all of these variables, treatment with PAH-specific therapy (HR 0.31, 95% CI 0.11–0.88, *p* = 0.03) was independently and significantly associated with decreased risk of mortality, and severely reduced DL_CO_ was independently and significantly associated with increased risk of mortality (HR 1.25, 95% CI 1.11–1.45, *p* = 0.048).

Associations with mortality among patients with lung disease and severe pulmonary hypertension were tested with multivariable Cox regression, with the dependent variable being mortality before a possible transplant. When accounting for all variables in the left-hand column in the regression model, PAH-specific therapy was independently and significantly associated with reduced risk of mortality, while severely reduced DL_CO_ was independently and significantly associated with increased risk of mortality.

## 4. Discussion

Among patients with chronic lung disease who met the new criteria for severe PH, those who received PAH-specific therapy had an increased survival probability. When accounting for the other pertinent clinical characteristics, treatment with PAH-specific therapy was independently and significantly associated with a 69% reduction in risk of mortality among patients with severe PH associated with lung disease. This benefit was not seen when including those with non-severe PH. 

The 2022 ESC/ERS guidelines for the definition and management of PH shifted the criteria for severe PH in chronic lung disease from an mPAP-based cutoff to a PVR cutoff, i.e., PVR > 5 WU. This was driven by studies of PH associated with COPD and ILD showing that PVR >5 WU was the best independent hemodynamic indicator of poor survival [7,8]. Similar findings between PVR and transplant-free survival were demonstrated in an international registry study of patients with PH associated with sarcoidosis [10]. Given that PVR > 5 WU has been shown to better predict survival outcomes, perhaps it predicts which patients will benefit from PAH-specific therapy, as seen in our study.

Gayen et al. (2023) had similar findings in a retrospective study that demonstrated improved survival with PAH-specific therapy in sarcoidosis-associated PH; all patients had pulmonary fibrosis due to sarcoidosis [11]. The treated group, on average, met the ESC/ERS definition of severe PH with a mean PVR of 6.6 WU compared to non-severe PH in the untreated group with a mean PVR of 3.8 WU. Despite having more severe baseline PH, those treated with PAH-specific therapy had improved survival probability. 

However, prior prospective studies investigating the benefit of pulmonary vasodilators in groups of patients with chronic lung disease and PH have had conflicting survival outcomes. In 2019, the RISE-IIP trial compared the use of riociguat versus placebo in patients with idiopathic interstitial pneumonia and PH but was terminated early given the increased mortality in the treatment group [12]. Later, the INCREASE trial demonstrated the superiority of inhaled treprostinil over a placebo for the six-minute walking distance (6MWD) and time to clinical worsening in patients with interstitial lung disease and PH during a 16-week study period [13]. This trial led to approval by the Food and Drug Administration (FDA) for inhaled treprostinil for group 3 PH. A post hoc analysis of the INCREASE trial used two statistical models to show increased long-term survival in patients who received treprostinil [14]. No trials to date have exclusively studied patients with chronic lung disease and severe PH. Notably, a recent meta-analysis was performed by analyzing PAH-specific therapy use in patients with COPD and severe PH, defined as mPAP > 35 mmHg or PVR > 5 WU; improvements in functional class and pulmonary hemodynamics were observed, but a comparison with untreated COPD-PH patients could not be performed to determine the potential mortality benefit [15].

Even when accounting for underlying lung disease, DL_CO_, 6MWD, hypoxemia, and BNP, PAH-specific therapy was still independently and significantly associated with reduced risk of mortality among patients with chronic lung disease and severe PH. In addition to PAH-specific therapy, DL_CO_ was associated with mortality among those with severe PH. This was consistent with prior studies that demonstrated reduced DL_CO_ to be predictive of worse outcomes in patients with various lung diseases and PH, as DL_CO_ is often reflective of PH severity [16,17,18]. It is interesting to note that in the severe PH cohort, similar survival was seen within the first 25 months when comparing those who received PAH-specific therapy to those who did not. Though it is difficult to determine exactly why, similar initial survival could be a result of the patient population; many of these patients were referred for lung transplant evaluation and, as such, had very close follow-up at our institution. Additionally, while these patients had advanced lung disease and PH, many were still functional enough to undergo the rigorous testing associated with lung transplant evaluation.

Unlike with PH associated with lung disease, randomized control trials have demonstrated a clear benefit of pulmonary vasodilator therapy for survival in all patients with PAH [19]. In a landmark trial by Barst et al. (1996), continuous intravenous epoprostenol significantly improved survival in PAH independent of the baseline 6MWD and led to the FDA approval of epoprostenol for PAH long before the INCREASE trial [20]. In 2005, a study of two trials and their extensions showed improved survival with oral bosentan in PAH patients, and in 2013, a trial with macitentan demonstrated significantly reduced mortality with treatment [21,22]. For PAH, FDA approval now exists for many pulmonary vasodilator drugs with multiple mechanisms of vasodilator action, with evidence demonstrating benefits with combined regimens.

Clinically, the distinction between PAH and PH associated with lung disease is the presence of chronic lung disease. Hemodynamically, these two groups share the same characteristics, except that, on average, patients with PAH tend to have more severe pulmonary hemodynamics than those of patients with PH associated with lung disease. The exception is a small percentage of patients who develop severe PH associated with lung disease [23,24]. The hallmark mechanism of PAH is pulmonary arterial intimal thickening. For PH associated with lung disease, the mechanism is not well understood but is proposed to be secondary to parenchymal destruction, resulting in the loss of pulmonary vasculature, dysfunctional pulmonary vasculature, and hypoxic vasoconstriction [25]. 

If parenchymal destruction were the central mechanism of severe PH associated with lung disease, then a linear relationship should exist between the burden of lung disease and PH. However, in our multivariate regression analysis, underlying lung disease and lung function were not specifically significantly associated with mortality among those with chronic lung disease and severe PH. Similarly, in a study of severe PH and COPD, the authors found that only 1.1% of patients in the cohort had severe COPD without other identifiable causes of PH, and the severity of PH was not associated with the severity of obstruction [23]. Additionally, a study of ILD patients demonstrated that the magnitude of restriction did not predict PH severity [26]. These findings have been replicated more recently, suggesting that patients with lung disease and severe PH must have an alternative mechanism of vascular remodeling unrelated to the extent of parenchymal involvement [27]. The results of our study add to a growing body of evidence that patients with severe PH associated with lung disease may have an alternative mechanism of PH that is more like that of PAH, where there is an established benefit of PAH-specific therapy for survival. 

There is also evidence that patients with severe PH associated with lung disease do not respond to hypoxia in the expected way with selective vasoconstriction. The main driver of pulmonary vasodilator intolerance in PH associated with lung disease was hypothesized to be the undoing of adaptive hypoxic vasoconstriction, leading to worse ventilation–perfusion mismatch. However, a recent study found that patients with COPD and severe PH had less obstruction and less hypoxic pulmonary vasoconstriction than those with moderate airflow obstruction and no PH, further suggesting that in patients with severe PH, pulmonary vascular dysfunction is independent of parenchymal destruction; in turn, these patients may benefit from PAH-specific therapy [28].

The strengths of our study are its large sample size and the variety of underlying lung diseases, as well as its novelty in utilizing the new ESC/ERS criteria for severe PH in chronic lung disease for both the classification and stratification of patients receiving PAH-specific therapy.

The main limitation of our study is that it is retrospective; patients received different classes of PAH-specific therapy at varying doses and sometimes in combined regimens. It is possible that when controlled for, a more nuanced effect may be seen, and as is seen in PAH, there may be a benefit to combined therapy over a single agent. Additionally, interventions such as how RHC was performed and how hemodynamic measurements such as PAWP were taken could not be standardized given the retrospective nature of the study. It is important to note that RHCs were performed on the patients’ baseline oxygen requirements, and hyperoxia can falsely reduce the mPAP and PVR in patients with PH. The methods for hemodynamic measurements themselves were also not protocolized. We did not include data on reduced right ventricular function in echocardiograms, though this has been associated with poor outcomes in similar patient cohorts. While this could be a limitation, reduced RV function is reflective of PH severity, which we captured with RHC data. Despite the lack of standardized methods for RHC and the ability to control for right ventricular dysfunction, the association with reduced risk of mortality with the use of PAH-specific therapy in those with lung disease and severe PH is still remarkable. 

We did not limit our study to exclusively pre-capillary PH, and a small percentage of patients with pre- and post-capillary PH were prescribed PAH-specific therapy, which is not in line with the treatment guidelines. However, while the inclusion of these patients may have added heterogeneity to our study sample, these patients were started on PAH-specific therapy because pre-capillary PH with lung disease was thought to be the driving mechanism of PH; their inclusion likely does not impact the significance of our findings.

Our study did not control for the use of lung-disease-specific therapy, such as antifibrotic medications, anti-inflammatory medications, and bronchodilators. It was not possible to control for the potential effects of these medications on survival due to the heterogeneity of lung diseases in this population. Moreover, given that our study was retrospective with patients who were cared for by various providers, we could not determine with certainty whether patients were on the optimal treatment, although we expected near-optimal treatment for the underlying lung diseases given the close follow-up necessary for the evaluation of transplants at our large academic center. Lastly, we were unable to control for type 2 disordered breathing, particularly in our patients with COPD, which may have impacted survival.

## 5. Conclusions

The latest ESC/ESR guidelines for the diagnosis and management of pulmonary hypertension shifted the definition of severe PH in chronic lung disease to PVR > 5 WU due to its prognostic efficacy in terms of mortality. We found that PAH-specific therapy may confer a mortality benefit in patients with chronic lung disease and severe PH defined as PVR > 5 WU, which is similar to the benefit of PAH-specific therapy in patients with PAH. It is notable that in our study, there was minimal use of parental prostacyclin analogs, which considerably worsen the quality of life due to the requirements of their use, yet there was still a significant survival advantage with PAH-specific therapy. Patients with chronic lung disease and severe PH may have similar pathophysiology to that of those with PAH; future randomized controlled trials are necessary to fully determine the potential beneficial effects of PAH-specific therapy in patients with chronic lung disease and severe PH. 

## Figures and Tables

**Table 1:** Demographic information.

	Received PAH-Specific Therapy (n = 123)	Did Not Receive PAH-Specific Therapy (n = 660)
Age, years (SD) *	61.2 (10.8)	63.8 (9.7)
Gender		
Male, n (%)	57 (46.3)	347 (52.6)
Female, n (%)	66 (53.7)	313 (47.4)
BMI, kg/m^2^ (SD)	29.3 (7.9)	29.0 (7.0)
Race		
White, n (%)	60 (48.8)	348 (52.7)
Black, n (%)	51 (41.5)	227 (34.4)
Hispanic, n (%)	10 (8.1)	64 (9.7)
Other, n (%)	2 (1.6)	21 (3.2)
Underlying lung disease *		
COPD, n (%)	35 (28.4)	294 (44.5)
IPF, n (%)	24 (19.5)	104 (15.8)
Other pulmonary fibrosis, n (%)	23 (18.7)	93 (14.1)
Non-fibrotic ILD, n (%)	7 (5.7)	29 (4.4)
Sarcoidosis, n (%)	22 (17.9)	48 (7.3)
CPFE, n (%)	12 (9.8)	92 (13.9)
Cardiac disease, n (%)	77 (62.6)	422 (63.9)
Chronic kidney disease, n (%) *	64 (52.0)	416 (63.0)
Diabetes, n (%)	37 (30.0)	224 (33.9)
Smoking history, n (%)	32 (26.0)	191 (28.9)

* *p* < 0.05. COPD: Chronic obstructive pulmonary disease. CPFE: Combined pulmonary fibrosis and emphysema. ILD: Interstitial lung disease. IPF: Idiopathic pulmonary fibrosis. PAH: Pulmonary arterial hypertension. SD: Standard deviation.

**Table 2:** Clinical characteristics at the time of right heart catheterization.

	Received PAH-Specific Therapy (n = 123)	Did Not Receive PAH-Specific Therapy (n = 660)
FEV1, L (SD)	1.4 (0.6)	1.3 (0.7)
FVC, L (SD)	2.0 (0.8)	2.1 (0.8)
Percent predicted DL_CO_ (SD)	27.6 (15.4)	28.1 (15.2)
mRAP, mmHg (SD)	7.9 (5.3)	7.1 (4.6)
sPAP, mmHg (SD) *	66.6 (19.5)	45.4 (13.9)
mPAP, mmHg (SD) *	41.1 (11.3)	29.3 (8.7)
mPAWP, mmHg (SD)	11.9 (5.6)	12.6 (11.4)
CO, L/min (SD)	4.7 (3.8)	5.1 (2.4)
CI, L/min/m^2^ (SD) *	2.3 (0.6)	2.6 (0.7)
PVR, WU (SD) *	8.3 (5.4)	4.4 (4.3)
Severe PH, n (%) *	88 (71.5)	158 (23.9)
6MWD, m (SD)	228.7 (97.1)	240.6 (95.7)
Oxygen requirements, L/min (SD)	6.2 (5.5)	5.5 (4.6)
BNP, pg/mL (SD) *	711.3 (413.9)	368.9 (276.7)

* *p* < 0.05. 6MWD: 6 min walking distance. BNP: Brain natriuretic peptide. CI: Cardiac index. CO: Cardiac output. DL_CO_: Diffusion capacity of carbon monoxide. FEV1: Forced expiratory volume in 1 s. FVC: Functional vital capacity. mPAP: Mean pulmonary artery pressure. sPAPP: Systolic pulmonary artery pressure. mPAWP: Mean pulmonary artery wedge pressure. PH: Pulmonary hypertension. PVR: Pulmonary vascular resistance. mRAP: Mean right atrial pressure. SD: Standard deviation. WU: Woods units.

**Table 3:** Outcomes in the entire cohort (n = 783).

	Received PAH-Specific Therapy (n = 123)	Did Not Receive PAH-Specific Therapy (n = 660)
Death, n (%)	28 (22.8)	112 (17.0)
Lung transplantation, n (%) *	47 (38.2)	383 (58.0)
Alive at last follow-up without a lung transplant, n (%) *	48 (39.0)	165 (25.0)
Median survival time from PH diagnosis, months (IQR)	98.5 (61.9, 135.2)	96.2 (90.4, 102.1)

* *p* < 0.05. IQR: Interquartile range. PH: Pulmonary hypertension.

**Table 4:** Clinical characteristics at the time of right heart catheterization in patients with severe pulmonary hypertension (n = 246).

	Received PAH-Specific Therapy (n = 88)	Did Not Receive PAH-Specific Therapy (n = 158)
FEV1, L (SD)	1.4 (0.6)	1.4 (0.6)
FVC, L (SD)	2.2 (0.9)	2.1 (0.8)
DL_CO_ < 40% predicted, n (%)	80 (90.9)	137 (86.7)
mRAP, mmHg (SD)	7.2 (5.9)	7.5 (4.9)
sPAP, mmHg (SD)	62.1 (17.5)	63.9 (17.9)
mPAP, mmHg (SD)	39.1 (11.6)	40.2 (10.3)
mPAWP, mmHg (SD)	11.1 (7.4)	11.3 (6.8)
CO, L/min (SD)	4.6 (2.7)	4.5 (2.7)
CI, L/min/m^2^ (SD)	2.2 (0.6)	2.3 (0.6)
PVR, WU (SD)	11.3 (10.7)	8.5 (4.2)
6MWD, m (SD)		
<150 m	17 (19.3)	40 (25.5)
150–300 m	54 (61.4)	89 (56.2)
>300 m	17 (19.3)	29 (18.3)
Hypoxemic, n (%)	77 (87.5)	128 (81.0)
BNP > 100 pg/mL, n (%) *	53 (60.2)	120 (75.9)

* *p* < 0.05. 6MWD: 6 min walking distance. BNP: Brain natriuretic peptide. CI: Cardiac index. CO: Cardiac output. DL_CO_: Diffusion capacity of carbon monoxide. FEV1: Forced expiratory volume in 1 s. FVC: Functional vital capacity. mPAP: Mean pulmonary artery pressure. sPAP: Systolic pulmonary artery pressure. mPAWP: Mean pulmonary artery wedge pressure. PH: Pulmonary hypertension. PVR: Pulmonary vascular resistance. mRAP: Mean right atrial pressure. SD: Standard deviation. WU: Woods units.

**Table 5:** Outcomes among patients with severe pulmonary hypertension (n = 246).

	Received PAH-Specific Therapy (n = 88)	Did Not Receive PAH-Specific Therapy (n = 158)
Death, n (%)	22 (25.0)	49 (31.0)
Lung transplantation, n (%) *	28 (31.8)	79 (50.0)
Alive at the last follow-up without a lung transplant, n (%) *	38 (43.2)	30 (19.0)
Median survival time from PH diagnosis, months (IQR) *	119.1 (68.0, 170.2)	48.9 (39.7, 58.0)

* *p* < 0.05. IQR: Interquartile range.

**Table 6:** Predictors of mortality in patients with chronic lung disease and severe pulmonary hypertension.

Variable	Association with Mortality
PAH-specific therapy	HR 0.31, 95% CI 0.11–0.88, *p* = 0.03 *
Underlying lung disease (COPD as reference)	
IPF	HR 0.46, 95% CI 0.15–1.44, *p* = 0.18
Other pulmonary fibrosis	HR 0.78, 95% CI 0.23–2.65, *p* = 0.70
Non-fibrotic ILD	HR 1.72, 95% CI 0.19–15.31, *p* = 0.63
Sarcoidosis	HR 0.38, 95% CI 0.10–1.40, *p* = 0.15
CPFE	HR 0.93, 95% CI 0.30–2.86, *p* = 0.90
FVC	HR 0.95, 95% CI 0.91–1.15, *p* = 0.68
DL_CO_ < 40% predicted	HR 1.25, 95% CI 1.11–1.45, *p* = 0.048 *
6MWD (compared to walking distance > 300 m)	
<150 m	HR 1.44, 95% CI 0.47–4.41, *p* = 0.52
150–300 m	HR 1.38, 95% CI 0.50–3.80, *p* = 0.53
Hypoxemia	HR 2.23, 95% CI 0.86–5.75, *p* = 0.10
BNP > 100 pg/mL	HR 0.92, 95% CI 0.41–2.09, *p* = 0.85

* *p* < 0.05. 6MWD: 6 min walking distance. BNP: Brain natriuretic peptide. CI: Confidence interval. COPD: Chronic obstructive pulmonary disease. CPFE: Combined pulmonary fibrosis and emphysema. DL_CO_: Diffusing capacity of the lungs for carbon monoxide. FVC: Functional vital capacity. HR: Hazard ratio. ILD: Interstitial lung disease. IPF: Idiopathic pulmonary fibrosis.

## Data Availability

The data that support the findings of this study are available from the corresponding author upon reasonable request.

## Supplementary Materials

The following supporting information can be downloaded at: https://www.mdpi.com/article/10.3390/jcdd11030089/s1, Table S1: Prescribed PAH-specific therapy.
