# Supplementary material for: Pulmonary Vasodilator Therapy Is Associated with Decreased Mortality in Patients with Chronic Lung Disease and Severe Pulmonary Hypertension
Source: J Cardiovasc Dev Dis. 2024 Mar 8;11(3):89. doi: 10.3390/jcdd11030089 (PMC10971116; doi:10.3390/jcdd11030089)
Supplement: Supplementary file 1 [file jcdd-11-00089-s001.zip › jcdd-2827092-supplementary.pdf]

Table S1. Prescribed Pulmonary Vasodilator Therapy.

Pulmonary Vasodilator Therapy	Number of Patients Prescribed (n=123)
PDE-5 inhibitor, n (%)	101 (82.1)
Endothelin Receptor Antagonist, n (%)	36 (29.3)
Inhaled Prostacyclin, n (%)	23 (18.7)
Subcutaneous Prostacyclin, n (%)	4 (3.3)
Intravenous Prostacyclin, n (%)	1 (0.8)
Oral Soluble Guanylate Cyclase Stimulator, n (%)	5 (4.1)

85 patients were prescribed single pulmonary vasodilator therapy, 30 patients were prescribed dual pulmonary vasodilator therapy, and 8 patients were prescribed triple pulmonary vasodilator therapy.
